# Balancing Rehabilitation Dose in Acute Stroke Decision-Making and Global Assessment (The BRIDGE Study)

**DOI:** 10.3390/jcm14196786

**Published:** 2025-09-25

**Authors:** Shinichi Watanabe, Wataru Yamauchi, Katsuma Shoka, Asahi Kawashima, Shogo Sawamura, Kousuke Kanamori, Tetsuya Furukawa, Yuji Naito, Naoki Takeshita, Keita Utiyama, Rtota Imai, Kanari Kiritani, Naoyuki Hashimoto, Hideaki Tanaka, Yushi Mitani, Takayuki Kitano, Daisuke Hori, Tatsuya Hayashi, Kenji Tsujimoto, Yasunari Morita

**Affiliations:** 1 Department of Physical Therapy, Faculty of Rehabilitation, Gifu University of Health Science, Gifu 500-8281, Japan; 2 Department of Rehabilitation Medicine, National Hospital Organization, Nagoya Medical Center, Nagoya 460-0001, Japan; krek.2978@gmail.com (K.K.); furutetsu13@gmail.com (T.F.); 3 Department of Rehabilitation, Gifu Prefectural Tajimi Hospital Gifu, Tajimi 507-8703, Japan; w.k20100801@gmail.com (W.Y.); asahi.kawashima10@gmail.com (A.K.); 4 Department of Rehabilitation, Central Japan International Medical Center, Minokamo 505-8510, Japan; s597katsu@gmail.com; 5 Department of Rehabilitation, Heisei College of Health Sciences, Gifu 501-1131, Japan; sawarm123@yahoo.co.jp; 6 Department of Rehabilitation, The National Hospital Organization Shizuoka Medical Center, Shimizu 411-0905, Japan; yujinaito.s60@gmail.com (Y.N.); 811616ny@gmail.com (N.T.); 7 Department of Rehabilitation, Japanese Red Cross Kanazawa Hospital, Kanazawa 921-8162, Japan; rsc57547@gmail.com; 8 Department of Rehabilitation, Municipal Ena Hospital, Ena 509-7201, Japan; l1zl1ij3tz@gmail.com; 9 Department of Rehabilitation, Japanese Red Cross Takayama Hospital, Takayama 506-8550, Japan; kanari999@yahoo.co.jp; 10 Department of Rehabilitation, Kanazawa University Hospital, Uchinada 920-0293, Japan; naohashimot531@gmail.com; 11 Department of Rehabilitation, Keiju Medical Center, Nanao 926-0816, Japan; hideaki.tanaka@keiju.co.jp; 12 Department of Rehabilitation, Japanese Red Cross Aichi Medical Center Nagoya Daini Hospital, Aichi 466-8650, Japan; ucdaft@gmail.com; 13 Department of Rehabilitation, Hamamatsu Medical Center, Hamamatsu 432-8580, Japan; kitano.hmc@gmail.com; 14 Department of Rehabilitation, Nakatsugawa Municipal Hospital, Nakatsugawa 508-8502, Japan; reha_hp@hosp.city.nakatsugawa.gifu.jp; 15 Department of Rehabilitation, Ichinomiyanishi Hospital, Ichinomiya 494-0001, Japan; mys5359@yahoo.co.jp (T.H.); tsujiken1260@gmail.com (K.T.); 16 Department of Emergency and Intensive Care Medicine, National Hospital Organization, Nagoya Medical Center, Nagoya 460-0001, Japan; moltlyme2@yahoo.co.jp

**Keywords:** stroke rehabilitation, early mobilization, mobilization quantification score, muscle atrophy, functional outcomes

## Abstract

**Background/Objectives:** Stroke remains a leading cause of disability in Japan, and early mobilization is an important strategy to prevent muscle atrophy and promote independence. However, the optimal intensity and duration of early rehabilitation remain unclear. This study aims to examine the association between rehabilitation dose during the acute phase of stroke and functional outcomes at 90 days post-onset. **Methods:** This multicenter prospective cohort study will enroll patients from twelve acute care hospitals across Japan, beginning in June 2026. Eligible patients are aged ≥ 18 years, expected to be hospitalized for ≥7 days, and initiated rehabilitation by day 2 after stroke onset. Rehabilitation dose will be quantified using the Mobilization Quantification Score (MQS). The primary outcome is functional status measured by the modified Rankin Scale (mRS) at 90 days. Secondary outcomes include muscle atrophy assessed by ultrasound, the Barthel Index, and physical performance measures. Subgroup analyses will evaluate how stroke severity modifies the dose–response relationship. **Results:** As this is a study protocol, results are not yet available. The study is designed to clarify the relationship between early rehabilitation dose and functional recovery after stroke. **Conclusions:** This is the first large-scale Japanese study to assess early stroke rehabilitation dosage using a standardized tool. Findings are expected to provide evidence for individualized, evidence-based mobilization strategies to optimize functional outcomes in stroke patients.

## 1. Introduction

Stroke is a leading cause of disability worldwide [1] and ranks fourth among the top ten causes of death in Japan. Although advances in medical science have improved survival rates for patients with severe stroke in recent years, the number of individuals living with long-term physical and cognitive sequelae has increased [2,3]. Among these, muscle atrophy in the paralyzed limbs of patients with hemiplegia, referred to as central atrophy, significantly affects both functional independence and quality of life [4]. In addition to stroke-related disability, critically ill patients may experience other complications, such as intensive care unit (ICU) acquired weakness, characterized by generalized weakness of the limbs and respiratory muscles, further impeding recovery [5,6]. Skeletal muscle atrophy in severely ill patients progresses rapidly by approximately 2% per day, amounting to a 12% reduction in muscle mass per week, and is directly associated with loss of functional independence and reduced quality of life. Therefore, early detection and prevention of muscle atrophy are crucial [7].

Rehabilitation is a primary strategy to promote functional recovery and enhance independence in stroke patients [8]. Once the patient’s neurological symptoms and vital signs stabilize during the acute phase, those with functional impairments transition to the post-acute phase and begin rehabilitation. Traditionally, active rehabilitation has been implemented during the post-acute phase, when patients are medically stable, with the goal of minimizing long-term disability [9]. More recently, increasing attention has been paid to early mobilization in critically ill patients. While several studies have reported the safety of early mobilization, its effectiveness remains a subject of debate [10]. Several systematic reviews and guideline papers have highlighted both the potential benefits and uncertainties of very early mobilization after stroke, noting variability in outcomes depending on patient severity, timing, and dose of interventions [11,12,13]. For example, one study reported that very early mobilization was associated with poorer functional outcomes and higher rates of adverse events at 3 months post-stroke compared to standard care [14]. However, a post hoc analysis of the same cohort revealed that patients who received shorter and more frequent mobilization sessions had significantly improved functional outcomes [15].

Previous research investigating the impact of early mobilization has examined patient outcomes in relation to age, stroke severity, and specific rehabilitation parameters, including intensity, duration, frequency, and timing of interventions [16,17,18]. It has also been suggested that optimal rehabilitation parameters should be tailored to the individual’s disease status and clinical characteristics [19]. Furthermore, considering both intensity and duration as components of physical activity may enable a more precise quantification of rehabilitation interventions, potentially leading to optimized clinical outcomes [13]. The acute phase of stroke is characterized by hemodynamic instability, rapid neurological deterioration, and accelerated skeletal muscle wasting, which can begin within hours of onset and progress by approximately 2% per day in severely ill patients [7]. During this period, patients are also at high risk for complications such as pneumonia, deep vein thrombosis, and pressure ulcers, all of which are exacerbated by immobility [9]. Therefore, timely initiation of rehabilitation is considered crucial not only to prevent secondary complications and muscle atrophy but also to maximize the potential for neurological recovery. This critical window highlights the need to carefully evaluate the appropriate dose and timing of early mobilization interventions. However, there is currently no consensus on the ideal dose, intensity, or timing of early mobilization for stroke patients in the acute phase. Each healthcare facility is thus exploring these parameters independently in pursuit of best practices [11]. Notably, there have been no large-scale studies in Japan evaluating the optimal daily dose of rehabilitation for stroke patients, nor have there been investigations focusing on the dosage of early-stage rehabilitation interventions.

Despite growing interest in early mobilization after stroke, key uncertainties remain. First, the optimal dose and timing in the acute phase are unresolved, with heterogeneous and sometimes conflicting results [14,15,20]. Second, most studies lack a standardized, quantitative dose metric, limiting cross-study comparability; the Mobilization Quantification Score (MQS) addresses this by integrating mobilization level and duration [16,21]. Finally, early-phase studies rarely incorporate objective muscle-atrophy assessments (e.g., rectus femoris ultrasound) despite prognostic relevance [7,22]. We hypothesize that the optimal rehabilitation dose during the acute phase of stroke varies by stroke severity: higher-intensity mobilization may promote greater independence in activities of daily living at 90 days post-stroke in patients with mild strokes, whereas lower-intensity mobilization may be more beneficial in those with severe strokes. We hypothesize that a higher early rehabilitation dose will be associated with better functional outcomes at 90 days. We further hypothesize that baseline stroke severity modifies this association: patients with mild-to-moderate stroke will benefit from higher MQS [21], whereas patients with severe stroke may benefit from moderate doses. Secondary hypotheses are that higher MQS will be associated with less rectus femoris muscle atrophy on ultrasound and better performance on physical assessment. To test this hypothesis, we will use the MQS to assess rehabilitation dose. In addition, we will analyze patient background characteristics, nutritional status, muscle atrophy, and differences in rehabilitation protocols across participating facilities. This study aims to establish standardized and optimized early mobilization practices and to evaluate the relationship between MQS-based rehabilitation dosage and functional independence in stroke patients.

## 2. Methods

This study protocol is reported in accordance with the Standard Protocol Items: Recommendations for Interventional Trials checklist (see Supplementary Materials Table S1).

### 2.1. Study Design

This is a multicenter, prospective, observational cohort study with a 90-day follow-up. The study will begin with the enrollment of the first patient and continue until the 90-day follow-up of the last enrolled participant. Twelve acute care hospitals across Japan will participate. Enrollment is scheduled to begin in June 2026 and continue for 18 months.

### 2.2. Ethical Approval

The study protocol has been approved by the Ethics Committee of Gifu Health Science University (approval number: 2025-012). All participating institutions will obtain approval from their respective ethics committees before patient recruitment. The study will be conducted in accordance with the Declaration of Helsinki and relevant Japanese ethical guidelines. Written informed consent will be obtained from all patients or their legally authorized representatives (e.g., next of kin) within 24 h of admission, if not already obtained during hospitalization. The study has been registered with the University Hospital Medical Information Network (UMIN000057876).

### 2.3. Study Setting

Twelve regional acute care hospitals in the Chubu region will participate (Supplementary materials Figure S1). Institutional characteristics (including the presence of unique clinical protocols, nurse-to-patient ratios, and availability of neurologists or other specialists) will be documented prior to enrollment and remain fixed throughout the study period. Rehabilitation practices vary among institutions but are based on standard references, including the Stroke Treatment Guidelines [20], Japanese Guidelines for Rehabilitation of Critically Ill Patients [23], and the Japanese Guidelines for Nutrition in Critically Ill Patients [24].

All hospitalized stroke patients will be screened within 24 h of admission by the research staff or departmental rehabilitation teams on working days. Eligible patients (if awake and cooperative) or their family members will be approached for consent within the first 24 h of admission, after which data collection will begin (Table 1).

### 2.4. Timeline

**Figure 1:**
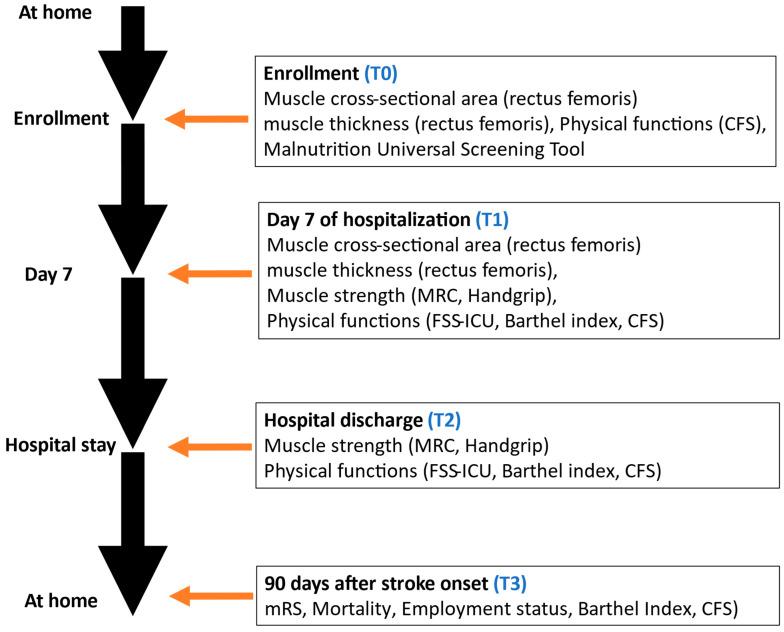
Flowchart of performed questionnaires during the study. Clinical Frailty Scale, CFS; Medical Research Council, MRC; Functional Status Score for the Intensive Care Unit, FSS-ICU.

After enrollment in the BRIDGE cohort, patients will remain in the study until loss to follow-up or completion of the 90-day assessment post-onset. Data will be collected at four time points: (1) enrollment, (2) day 7 of hospitalization, (3) hospital discharge, and (4) 90 days after stroke onset (Figure 1).

### 2.5. Participants

a. Inclusion criteria:

1. Patients diagnosed with acute stroke, specifically including ischemic stroke (cerebral infarction) and hemorrhagic stroke (intracerebral hemorrhage), who are expected to be hospitalized for ≥7 days. Patients with subarachnoid hemorrhage or transient ischemic attack (TIA) will be excluded because their rehabilitation course differs substantially from that of ischemic and hemorrhagic stroke;
2. Age ≥ 18 years;
3. Provision of informed consent;
4. Initiation of rehabilitation by day 2 of admission.

b. Exclusion criteria:

1. Pre-hospitalization mRS ≥ 3 (unable to walk even with aids);
2. Terminal care patients or those with non-curative intent;
3. Patients with anticipated prolonged immobility due to trauma (e.g., multiple unstable fractures, burns, amputations);
4. Inability to communicate in Japanese;
5. Explicit refusal to allow use of clinical data for research.

c. Anticipated cohort characteristics and planned reporting.

Based on stroke case-mix in the participating hospitals, we anticipate a sex distribution of approximately 55–60% male and 40–45% female, with a mean age around 70 years. For transparency and comparability, we predefine age strata as <65, 65–79, and ≥80 years. Baseline characteristics will be summarized overall and, where appropriate, by sex and age group.

### 2.6. Primary Outcome

The primary outcome is the modified Rankin Scale (mRS) score at 90 days post-stroke. The mRS ranges from 0 (no symptoms) to 6 (death) and is the most widely used functional outcome measure in stroke trials [25]. An mRS ≤ 2 at 90 days is defined as a favorable outcome (Table 2).

### 2.7. Secondary Outcomes

As secondary outcomes, we first selected the item of change in muscle atrophy using ultrasound examination [7,22]. This will allow us to determine the optimal MQS dose for muscle atrophy results. Next, we selected the items of the Barthel Index [26], Medical Research Council score [27], functional status score in ICU [28], grip strength [29], Clinical Frailty Scale [30], return to work, and complications. We aim to investigate these items and quantify ICU-acquired weakness from multiple perspectives (Table 2). We will also record adverse events during the rehabilitation intervention (falls, cardiac arrest, tachycardia > 130 bpm, systolic blood pressure < 80 or >200, ventricular tachycardia, other dangerous arrhythmias, arterial oxygen saturation of pulse oximetry < 80% for >3 min, and other device removal).

### 2.8. Baseline Characteristics and Treatment

The baseline characteristics of enrolled patients will be prospectively collected, including baseline factors such as age, height, weight, handedness, employment status, existing comorbidities, pre-admission mRS, and frailty; disease factors such as culprit vessel, lesion, and national institutes of health stroke scale [31]; biochemical data such as C-reactive protein, lymphocyte count, and serum albumin; and nutrition factors such as the Malnutrition Universal Screening Tool [32] at admission. The National Institutes of Health Stroke Scale will be recorded at admission, day 7, and at discharge. Details of treatments that may affect outcomes, such as surgery, use of Tissue Plasminogen Activator, noninvasive ventilation, mechanical ventilation, and tracheostomy, will also be prospectively collected.

### 2.9. Data Source/Measurements

At each of the above time points, ultrasound will be used to evaluate the cross-sectional area and muscle thickness of the rectus femoris. All collected data will be prepared according to standard protocols published in the field and analyzed by a team experienced in muscle ultrasound (physiotherapists and/or neurologists and rehabilitation physicians currently working in the hospital).

### 2.10. Ultrasonography Assessment

Ultrasound will be used to measure the rectus femoris muscle cross-sectional area and thickness in supine patients with extended knees. Measurements will be taken at the anterior superior iliac spine and the distal third of the thigh using B-mode with a linear probe. For consistency, anatomical landmarks will be marked for repeated measures. If one image cannot capture the entire muscle, it will be reconstructed from multiple sections. Each measurement is taken three times, and the median value is used. Pre-study training ensures that intra- and inter-rater variation remains below 3%.

### 2.11. Rehabilitation Protocol

Participants in the BRIDGE cohort will receive their usual rehabilitation at their respective institutions. We aim to exercise all participants equally every day, based on a five-stage protocol (Level 1: passive range of motion and respiratory physiotherapy, Level 2: active range of motion, Level 3: sitting exercise, Level 4: standing exercise, Level 5: walking exercise) tailored to each participating hospital. In addition, adverse events during implementation will also be indicated with appropriate values in the categories of medical, cardiovascular, respiratory, and neurological problems. In case of deviation from these values, the patient will be immediately placed on bed rest, and the event will be considered an adverse event. After level 5 is achieved, a physiotherapist or occupational therapist will provide each patient with rehabilitation, including muscle strengthening, balance, walking, and stair climbing, for at least 20 min on weekdays, according to the rehabilitation policy of each hospital.

### 2.12. Data Management and Follow-Up

Patient data will be managed in compliance with Japanese data protection guidelines. A unique study number will be assigned upon enrollment. Follow-up assessments will be conducted via telephone at 90 days post-onset, collecting data on survival, employment, and mRS. If unreachable after multiple attempts, patients will be considered lost to follow-up. Informed consent includes potential secondary use of anonymized data, contingent on new ethical approval.

### 2.13. Rehabilitation Dose

Rehabilitation dose is defined using the MQS over days 1–7 post-admission. MQS combines mobilization level (assessed using the ICU mobility scale [33]) and duration (measured with a stopwatch). Only active mobilization time is included; preparation or rest is excluded. The MQS is averaged across the first 7 days to reflect the daily rehabilitation dose (Japanese version MQS [21]. It should be noted that rehabilitation itself continues beyond the first 7 days until hospital discharge, according to each institution’s standard practice. After discharge, patients typically continue rehabilitation in post-acute care facilities or outpatient/community settings, depending on their medical condition and regional healthcare resources. Functional outcomes at 90 days will therefore reflect the combined effect of early inpatient rehabilitation and subsequent standard care.

### 2.14. Statistical Methods

There is no maximum sample size for this study; however, the outcome may be subject to targets or maximum sample sizes, which will be specified in the relevant sub-protocol. The expected sample size is ≥200 patients (minimum 10 patients/facilities, ≥20 facilities) to provide a sufficient number of different rehabilitation doses to predict functional outcomes and build robust models for protocol use. To avoid over-representation of some centers, data collection is limited to 50 patients per facility. Patient enrollment will therefore not be strictly equal across hospitals; each facility is expected to enroll at least 10 patients, with a maximum of 50 patients, depending on their clinical volume. This strategy ensures diversity in practice patterns while preventing over-representation of any single center. The number of enrolled patients and facilities is a sufficient sample size to capture the various variations in practice and treatment. It is considered feasible to collect a sample of this calculated size because each participating facility should be able to enroll at least one patient per month based on the number of stroke patients hospitalized at each facility in the past.

Baseline characteristics and outcomes will be summarized using descriptive statistics. Continuous variables will be presented as means with standard deviations (SDs) or medians with interquartile ranges (IQRs), while categorical variables will be presented as frequencies and percentages (*n*, %).

The primary analysis will examine the association between the mean rehabilitation dose (MQS score) during the first 7 days of hospitalization and the primary outcome of a favorable functional outcome (mRS ≤ 2) at 90 days. This association will be evaluated using a multivariable logistic regression model. The model will be adjusted for a pre-specified set of covariates selected for their established prognostic significance, including age, sex, pre-admission mRS, baseline National Institutes of Health Stroke Scale (NIHSS) score, stroke subtype (ischemic or hemorrhagic), and participating facility (as a random effect).

For secondary outcomes, multivariable linear regression models will be used for continuous variables, such as the Barthel Index. A pre-specified subgroup analysis will be conducted to explore whether the effect of rehabilitation dose is modified by initial stroke severity. An interaction term between the MQS score and the severity category (defined by baseline NIHSS) will be introduced into the primary model.

Time-to-event outcomes will be analyzed using Kaplan–Meier curves and Cox proportional hazards regression models, adjusted for baseline characteristics. For survival analysis, patients will be stratified into groups based on quartiles of rehabilitation volume and pre-defined stroke severity categories. Missing data for covariates will be handled using multiple imputation under the missing-at-random assumption. The primary analysis will be based on the pooled results from the imputed datasets, with a complete-case analysis performed as a sensitivity analysis.

All statistical analyses will be performed using JMP (version 13.0; SAS Institute, Cary, NC, USA) and IBM SPSS software (version 23.0; IBM, Armonk, NY, USA). A two-sided *p*-value of <0.05 will be considered statistically significant.

## 3. Discussion

This multicenter prospective cohort study aims to evaluate the association between rehabilitation dose quantified using MQS and functional outcomes in patients with acute stroke. The study is designed to address the current lack of evidence regarding optimal early mobilization parameters, particularly in the acute phase of stroke care. While early rehabilitation has been widely promoted in recent years, clinical practices remain variable, with no established consensus on ideal dose, timing, or intensity [34]. Our study is the first large-scale investigation in Japan to quantitatively assess daily rehabilitation dose using a validated measure and its relationship with long-term independence in activities of daily living.

Prior studies on early mobilization in stroke patients have yielded inconsistent findings. For instance, while very early mobilization was associated with poorer outcomes in some randomized controlled trials [14], further post hoc analyses have suggested that mobilization delivered in shorter, more frequent sessions may improve recovery [15]. These conflicting findings may be attributed to differences in rehabilitation intensity, timing, patient severity, and methodological inconsistencies across studies [12]. By incorporating MQS, which integrates both mobilization level and duration, our study provides a more comprehensive and standardized approach to measuring rehabilitation exposure [16]. Furthermore, by stratifying patients based on stroke severity, our study will allow exploration of differential responses to rehabilitation dose, which may help define individualized, severity-specific strategies.

Another strength of this study is the integration of multiple functional, physical, and clinical outcomes, including mRS, Barthel Index, Medical Research Council score, muscle ultrasound, and frailty assessments, which enables a multifaceted evaluation of recovery. The inclusion of ultrasound to objectively assess atrophy of the rectus femoris provides novel insight into the impact of early mobilization on muscle preservation, which has been relatively underexplored in stroke rehabilitation research [35,36]. Additionally, our protocol controls for key patient-level and institutional factors, including comorbidities, nutritional status, rehabilitation policy, and institutional staffing, enhancing the robustness of our multivariate analyses.

By quantifying early rehabilitation dose with MQS over days 1–7 and linking it to 90-day outcomes, this protocol is designed to yield directly actionable outputs for practice. First, clinicians can translate MQS into severity-specific dose targets during daily rounds, facilitating individualized prescriptions rather than one-size-fits-all approaches. Second, routine rectus femoris ultrasound enables early detection of muscle atrophy and dose adjustment when deterioration is observed. Third, aggregating MQS and outcomes at the facility level provides benchmarking metrics that support quality-improvement cycles and alignment across centers. Finally, these data can inform guideline refinement and the design of future randomized trials by identifying dose bands and patient subgroups most likely to benefit.

Nevertheless, some limitations must be acknowledged. First, as an observational study, causal relationships cannot be definitively established. Second, rehabilitation practices are not standardized across facilities, which may introduce variability. However, this also enhances external validity by reflecting real-world conditions. Finally, the use of telephone interviews for follow-up assessments may be susceptible to recall bias, although structured questionnaires and clear documentation (e.g., who assisted in responses) aim to minimize this limitation [37].

## 4. Conclusions

This study will be the first multicenter prospective cohort study in Japan to investigate the association between rehabilitation dose and functional outcomes in patients with acute stroke using a standardized measure, the MQS. By exploring dose–response relationships stratified by stroke severity and incorporating multiple physical and functional indicators, the study seeks to identify optimal rehabilitation strategies that are individualized, evidence-based, and implementable in real-world clinical settings. The findings of this study are expected to contribute to the standardization of early rehabilitation protocols and to improve long-term outcomes for stroke patients across diverse healthcare environments.

## Figures and Tables

**Table 1:** Schedule of enrolment, interventions, and assessments.

	Enrolment	Post-Allocation		Close-Out
TIMEPOINT	Hospital Admission T0	Day 7 T1	Hospital Discharge T2	After Day 90 T3
**ENROLMENT**				
Eligibility screen	×			
Informed consent	×			
Cohort enrollment (index date)	×			
**ASSESSMENTS:**				
Baseline variables	×			
Muscle cross-sectional area muscle thickness	×	×		
Physical functions (Clinical Frail Scale)	×	×	×	×
Physical functions (Functional status score-ICU, Barthel index)		×	×	
Muscle strength (Medical Research Council, Handgrip)		×	×	
mRS	×	×	×	×
Exposure ascertainment (MQS; mean of Days 1–7)		×	×	

Abbreviations: ICU; intensive care unit, mRS; modified Rankin Scale, MQS; Mobilization Quantification Score.

**Table 2:** Details of outcome measures at follow-up.

Variable	Description
Survival	If a patient dies during follow-up, date of death is recorded
Employment status	Whether the patient/family has a job at follow-up (full-time or part-time) and whether the job is the same as before hospital admission
General information	Readmission to hospital during follow-up
National Institutes of Health Stroke Scale	The National Institutes of Health Stroke Scale is a 15-item impairment scale, intended to evaluate neurologic outcome and degree of recovery for patients with stroke. The scale assesses level of consciousness, extraocular movements, visual fields, facial muscle function, extremity strength, sensory function, coordination, language, speech, and hemi-inattention.
Physical function/activities of daily living	
mRS	The mRS ranges from 0 (no symptoms) to 6 (death) and is the most widely used functional outcome measure in stroke trials. An mRS ≤ 2 at 90 days is defined as a favorable outcome.
Clinical Frail Scale	The Clinical Frailty Scale is a judgment-based frailty tool that evaluates specific domains, including comorbidity, function, and cognition, to generate a frailty score ranging from 1 (very fit) to 9 (terminally ill).
FSS-ICU	The FSS-ICU is a performance-based measure that assesses physical function in critically ill patients. It evaluates five tasks: (1) rolling, (2) supine to sit transfer, (3) sitting at the edge of the bed, (4) sit to stand transfer, and (5) walking. Each task is scored from 0 (unable to perform) to 7 (complete independence), for a total score ranging from 0 to 35. Higher scores indicate better physical function.
MRC score	The MRC score is a bedside tool used to assess muscle strength in critically ill patients. It evaluates six muscle groups bilaterally: shoulder abduction, elbow flexion, wrist extension, hip flexion, knee extension, and ankle dorsiflexion. Each muscle group is graded on a scale from 0 (no contraction) to 5 (normal strength), yielding a total score ranging from 0 to 60. A score below 48 typically indicates clinically significant muscle weakness.

mRS; modified Rankin Scale, MRC score; Medical Research Council score, Functional Status Score for the ICU; FSS-ICU, MUST; Malnutrition Universal Screening Tool.

## Data Availability

No new data were created or analyzed in this study.

## Supplementary Materials

The following supporting information can be downloaded at: https://www.mdpi.com/article/10.3390/jcm14196786/s1, Table S1: SPIRIT Checklist for Trials; Figure S1: Participating hospitals; Table S2: List of Collaborators.

## Abbreviations

The following abbreviations are used in this manuscript:

ICU	Intensive Care Unit
mRS	Modified Rankin Scale
MQS	Mobilization Quantification Score
CFS	Clinical Frailty Scale
FSS-ICU	Functional Status Score for the Intensive Care Unit
MRC	Medical Research Council (score)
NIHSS	National Institutes of Health Stroke Scale
UMIN	University Hospital Medical Information Network
MUST	Malnutrition Universal Screening Tool
SD	Standard Deviation
IQR	Interquartile Range
SPIRIT	Standard Protocol Items: Recommendations for Interventional Trials
